# Affect and gaze responses during an Emotion-Evoking Task in infants at an increased likelihood for autism spectrum disorder

**DOI:** 10.1186/s13229-021-00468-0

**Published:** 2021-10-06

**Authors:** Lori-Ann R. Sacrey, Lonnie Zwaigenbaum, Jessica A. Brian, Isabel M. Smith, Vickie Armstrong, Sarah Raza, Tracy Vaillancourt, Louis A. Schmidt

**Affiliations:** 1 Department of Pediatrics, Autism Research Centre – E209, Glenrose Rehabilitation Hospital, University of Alberta, 10230-111 Avenue, Edmonton, AB T5G 0B7 Canada; 2 Bloorview Research Institute, University of Toronto, Toronto, ON Canada; 3 IWK Health Centre, Dalhousie University, Halifax, NS Canada; 4 University of Ottawa, Ottawa, ON Canada; 5 McMaster University, Hamilton, ON Canada

**Keywords:** Emotion regulation, Affect, Gaze, Temperament, Autism*, Increased likelihood cohort

## Abstract

**Background:**

The majority of research examining emotional difficulties in autism spectrum disorder (ASD) prior to age 2 relies on parent report.

**Methods:**

We examined behavioral responses (affect and gaze) during emotionally salient tasks designed to elicit mildly positive and negative emotional states in infants. At 12 and 18 months, infants at an increased likelihood for an ASD diagnosis (IL; have an older sibling with ASD; *n* = 60) and low likelihood (LL; no family history of ASD; *n* = 21) completed the Emotion-Evoking (EE) Task and parents completed the Infant Behavior Questionnaire-Revised (IBQ-R). All children received an Autism Diagnostic Observation Scale—second Edition assessment for ASD symptomatology at 24 months.

**Results:**

The main findings were (1) the IL group displayed higher rates of negative affect and spent less time looking at the task objects compared to the LL group, and (2) affect and gaze scores at 12 and 18 months, but not scores on the IBQ-R, predicted ASD symptoms at 24 months.

**Limitations:**

The data were drawn from an IL sample and may not be generalizable to the general ASD population, and the children were not followed to determine a diagnosis of ASD.

**Conclusion:**

These results suggest that behavioral responses can provide important information that complements parent reports of emotional regulation in IL infants as early as 12 months of age.

**Supplementary Information:**

The online version contains supplementary material available at 10.1186/s13229-021-00468-0.

## Background

Emotional regulation (ER) begins to appear in the first year of life and refers to the ability to modulate the occurrence, intensity, and valence of emotional reactions through intrinsic (learned with experience) and extrinsic (with assistance from others) strategies [9,46,59, 26]. Depending on context, ER can be unconscious or conscious, controlled or automatic, and extrinsic (e.g., parent regulating child’s emotions) or intrinsic (child regulating own emotions) [27]. Emotional regulation is predictive of several domains of development in childhood, including behavioral problems (e.g., externalizing behaviors, [49, 63], social skills [14, 15], and academic skills [5, 65]). Difficulties in ER also show high concordance with the core features of autism spectrum disorder (ASD, [43, 50, 61]). For example, impairments in communication, affective expression, and reciprocal play are often associated with emotional dysregulation [10]. Although neurotypical children make developmental strides in learning to regulate their emotions during their early school years, many children with neurodevelopmental disorders, including those on the autism spectrum, continue to struggle with ER into adolescence and adulthood [45].

Emotional regulation can be measured during childhood using questionnaires, direct observation, and physiological measurement, such as heart rate [64]. Studies of ER in individuals with ASD suggest that they experience increased negative emotions and reduced positive emotions [3,7,56, 30]. Most previous research examining ER in *very young* children (2 years and under) has used parent questionnaires [44] that assess temperament, that is, individual differences in reactivity and self-regulation of emotion, attention, and activity [53], rather than direct (i.e., physiological) measures. For example, Capps et al. [7] compared ratings on the parent-rated Emotion Behavior Checklist [33] between children with ASD and neurotypical children who were matched on mental age (24 months). Parents of children with ASD rated their children as showing more sadness and fear, as well as less joy than did parents of neurotypical children. Similarly, Garon et al. [20] examined parent ratings on the Infant Behavior Questionnaire-Revised (IBQ-R, [52]) at 12 months and the Toddler Behavior Assessment Questionnaire-Revised [54] at 24 months and found that parents of infants at an increased likelihood of an ASD diagnosis (IL, younger siblings of children diagnosed with ASD) rated their children as showing higher levels of fear, sadness, and anger, and lower inhibitory control, soothability, attention focus, high pleasure, and low pleasure compared to typically developing peers. Furthermore, IL infant siblings who were later diagnosed with ASD at age 3 showed lower levels of positive affect at 12 and 24 months and lower effortful control at 24 months, compared to IL infant siblings who were not diagnosed with ASD at age 3, Garon et al. [20]. Most recently, Ersoy et al. [16] asked parents of IL and children without a family history of ASD (low likelihood, LL) children to complete the IBQ-R at 9 and 15 months of age, when no group differences emerged for the sadness scale. However, the Early Childhood Behavior Questionnaire [51] administered at 24 months yielded higher levels of sadness among the IL group than for LL children.

The earliest age at which the emotional expressivity of children with ASD has been *directly observed* during emotionally valanced tasks was two years. Macari et al. [39] found that children at age 2 with ASD displayed lower intensity fear, but no differences for anger or joy when compared to age-matched neurotypical children. In the only other study to look at observed emotion, videos taken at 12 months during toy play (not designed as an emotionally salient task) showed that children later diagnosed with ASD had lower rates of positive affect (i.e., smiling) compared to children who were not diagnosed with ASD [17]. Thus, further examination of positive and negative emotional responses early in life in relation to ASD is warranted.

In the present study, we examined behavioral responses to emotionally salient stimuli at 12 and 18 months of age in children who were at a low likelihood (LL; no family history of ASD) and IL (infant sibling of child with ASD) for ASD. Predictions were informed by previous studies of ER in older children with ASD [3,7,56, 30]. Specifically, we predicted that (1) children in the IL group would display higher levels of negative affect and lower levels of positive affect during the Emotion-Evoking (EE) Task, which was adapted from the Laboratory Temperament Assessment Battery (Lab-TAB, Goldsmith and Rothbart 1996), compared to children in the LL group at 12 and 18 months; and (2) affect and gaze at 12 and 18 months would predict ASD symptoms at 24 months. To test the assumption that our EE task was a valid measure of ER, we predicted that affect and gaze would be associated with concurrent ratings on the IBQ-R at 12 and 18 months.

## Method

### Participants

Infant siblings of children with ASD were recruited between the ages of 6 and 12 months from families attending one of three multidisciplinary ASD clinical centers and surrounding communities [locations blinded]. Participants were assessed at 12, 18, and 24 months of age. The research ethics board at each institution approved this study, and all families gave written informed consent prior to study enrollment.

For the IL group, diagnosis of ASD in the older sibling (i.e., proband) was confirmed by a review of diagnostic records, using DSM-5 [1] criteria. The IL infants did not have identifiable neurological or genetic conditions, nor severe sensory or motor impairments. LL infants were recruited from the same communities, had at least one older sibling but no reported first- or second-degree relatives with an ASD diagnosis. All participants were born at 36–42 weeks of gestation, with birth weight greater than 2500 g.

### Emotion-Evoking (EE) Task

Positive and negative affect, as well as gaze, was measured using tasks adapted from the Laboratory Temperament Assessment Battery (Lab-TAB; [24]), a comprehensive temperament assessment that includes episodes designed to elicit behavior related to differing dimensions of temperament, including smiling, reaching, crying, touching, or changes in facial expression. The EE Task was completed at 12 and 18 months of age.

#### EE task set-up

**Fig. 1:**
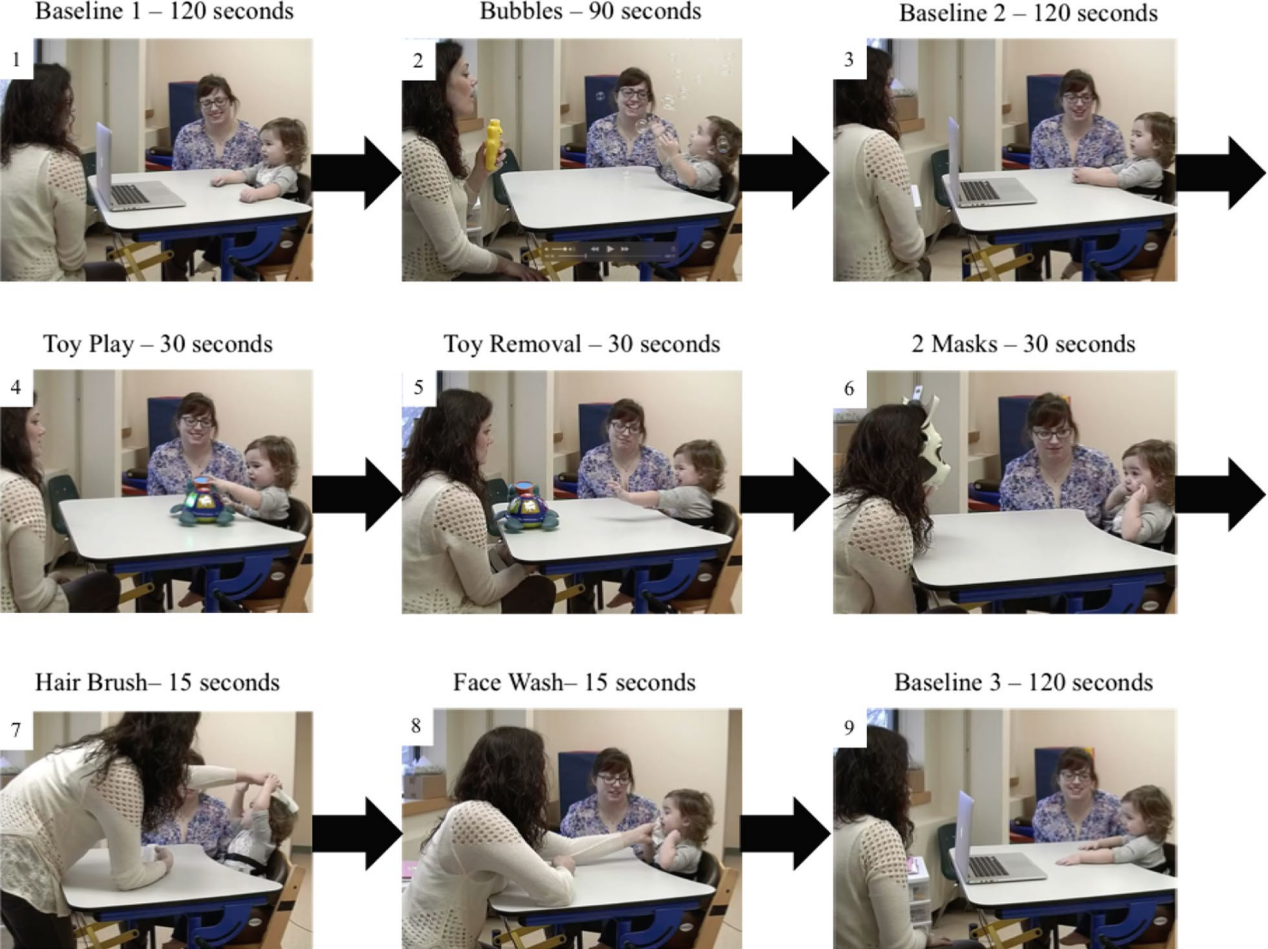
Emotion-Evoking Task

Children were seated at a height-adjustable table in a high-chair with their parent seated to their right. As there are no general instructions regarding where the parent should be seated with respect to the child, we used the parent location guidelines for the mask and toy removal tasks in the Lab-TAB manual [24]. All phases of the EE Task, including the Baseline video, occurred with the child seated in the high-chair. The Baseline video was shown on a laptop or computer monitor, which was placed on the table in front of the child (see Fig. 1). Once the video ended, the computer/monitor was placed on the floor next to the examiner and out of sight of the child. The objects used for each task were held in an opaque bin next to the examiner and out of the child’s sight. The phases included within our EE Task are shown in Fig. 1:

1. *Baseline 1 phase *Child was shown a 2-min video comprising 15-s clips of intermixed screensaver images and ‘Baby Einstein’ clips accompanied by instrumental music to allow an opportunity to acclimate to the research setting (neutral task).
2. *Bubbles phase* Experimenter blew bubbles towards child and directed child’s attention toward bubbles for 90 s (positive task).
3. *Baseline 2 phase *Child was shown the same 2-min video from Baseline 1 to allow an opportunity to return to baseline (neutral task).
4. *Toy Play phase* Child was given a toy that lights up and makes musical noise when its buttons are pushed, for 30 s (positive task).
5. *Toy Removal phase* Appealing toy (used in Toy Play) was placed out of reach, but within sight of child for 30 s (negative task).
6. *Masks phase* Experimenter wore a blank mask on their face and sat still and quiet for 15 s, followed by wearing a cow mask and sitting quietly for 15 s (negative task).
7. *Hair Brushing phase* Experimenter brushed child’s hair with comb or soft brush for 15 s (negative task).
8. *Face Washing phase* Experimenter gently washed child’s face (forehead, cheeks, chin, nose) with baby wipe for 15 s (negative task).
9. *Baseline 3 phase *Child watched the same 2-min video from Baselines 1 and 2 to allow an opportunity to return to baseline following the negative tasks.

#### Affect and gaze coding

The EE Task was video-recorded, and affect and gaze were coded off-line from video-recordings using Noldus Observer 13 XT behavioral coding software (see Additional file 1: Table S1 for brief coding scheme). Coding was completed in two separate runs/viewings of the entire video-recording for each participant; once for phase (onset and offset) and affect, and separately for gaze. Videos were played at real time for coding. Phases were coded continuously, and codes were mutually exclusive and exhaustive, such that one code ended the previous code. Periods between phases were coded as ‘transition’ episodes and were not coded for behavior or included in analyses.

##### Affect

Affect was coded in 5-s intervals as either negative, neutral, or positive on a 5-point scale from − 2 to + 2, based on both facial and vocal cues. Periods during which the face was not visible and vocal cues for affect were absent were coded as ‘not codable’ (for definitions associated with use of facial or vocal cues alone to code affect, see Additional file 1). Interval coding was selected because onset and offset of affect intensity were difficult to define and facial affect cues can change rapidly. The variable for mean affect was calculated for each phase of the EE Task by taking the mean of means of the 5-s intervals. For example, the Masks phase was 30 s and comprised 6 coded intervals (each interval was 5 s). The mean affect for the Masks phase was calculated as the sum of the codes for each of the 6 intervals divided by 6.

##### Gaze

Gaze was coded continuously (as opposed to interval coding), and codes were mutually exclusive and exhaustive. The types of behavior of interest included infant *looking* at the ‘on-task’ object, ‘off-task’ objects, the experimenter conducting the task, the parent sitting beside the child, and gaze aversion. Off-task objects included objects that were proximal to the infant that the infant manipulated or interacted with (e.g., sensors and cables, as well as objects that parents may have given their children unexpectedly, such as toys or sippy cups, which were removed as quickly as possible). ‘Other’ was used to code any other looking behavior (e.g., scanning the room). The data included in this paper assessed the *on-task* gaze behavior only. The *on-task* gaze objects were the computer monitor for the baseline phases, bubbles or bubble wand for Bubbles phase, the toy used for the Toy Play and Toy Removal phases (same toy), the two masks used in Masks phase, the comb/brush used in Hair Brushing phase, and the baby wipe used in Face Washing phase. The variable for percentage of time spent on the “on-task” object was calculated for each phase of the EE Task using the following formula:

$$\left[ {\frac{{{\text{time}}\;{\text{spent}}\;{\text{looking}}\;{\text{at}}\;{\text{``on}}\;{\text{task"}} \;{\text{object}}}}{{{\text{length}}\;{\text{of}}\;{\text{phase}}}}} \right] \times 100$$

#### Inter-rater reliability

Two raters coded 20% of the videos to assess for reliability. Inter-rater reliability was assessed using Cohen’s kappa (*κ*), with 0.01–0.20 representing no to slight agreement, 0.21–0.4 representing fair agreement, 0.41–0.60 as moderate agreement, 0.61–0.80 representing substantial agreement, and 0.81–1.00 representing almost perfect agreement [41]. The formula is

$$\kappa = \frac{{p_{{\text{o}}} - p_{{\text{c}}} }}{{1 - p_{{\text{c}}} }}$$

where *p*_o_ is the observed proportion of agreements and *p*_*c*_ is the proportion of agreements expected by chance [8]. For affect, *κ* = 81% when assessing for no differences in code value (both raters gave the same code). When reliability was assessed using a modifier margin of 1 (codes were within ± 1 point), *κ* = 95% was achieved. For gaze, *κ* = 89% was achieved when calculating the percentage agreement for duration of gaze codes for the two raters. The raters were blind to group membership, with the exception that the reliability rater was involved in study visits at one site but remained blind to enrollment group (IL vs. LL) and ASD symptom history.

### Infant behavior questionnaire-revised (IBQ-R)

The IBQ-R [52] was designed to assess temperament in children aged 3–12 months and has fourteen subscales: activity level, smiling and laughing, fear, distress to limitations, high pleasure, low pleasure, soothability, falling reactivity, cuddliness, sadness, approach, vocal reactivity, perceptual sensitivity, and duration of orienting. Items are rated on a 7-point scale ranging from 1 (never) to 7 (always), with an 8th option for ‘does not apply’. Calculation of the mean ratings on all items in a particular scale, minus the ‘does not apply’ items, yields scaled scores. The IBQ-R can be completed by parents within 15 min and is well-validated and has excellent test-retest reliability [23]. Cronbach’s alpha for the 14 subscales of the IBQ-R ranged from .76 to .93 at 12 months and .71 to .91 at 18 months for our sample (see Additional file 1: Table S2).

We chose to have parents complete the IBQ-R at both the 12- and 18-month visits, rather than the Early Childhood Behavior Questionnaire (ECBQ) (for children between 18 and 36 months [51]) at the 18-month visit for three reasons. First, we wanted to use the same measure at both 12 and 18 months of age to compare to the EE Task. Second, social-emotional development follows an expected trajectory in the first 12–18 months of life [40], which can be influenced by ASD [38]. Third, many children with ASD have lower mental ages than their typically developing counterparts, which can affect performance on behavioral assessments and questionnaires [29]. Developmental age equivalencies in our sample were assessed using the Mullen Scales of Early Learning [47], and scores on the IBQ-R subscales were correlated to determine relatedness in scoring.

### Mullen scales of early learning (Mullen)

The Mullen [47] is a developmental measure that assesses Visual Reception, Receptive Language, Expressive Language, Fine Motor and Gross Motor abilities and has an Early Learning Composite comprising the first four scales. We administered the Mullen at 12 and 18 months to assess developmental age equivalencies in our sample.

### Autism diagnostic observation schedule -2nd edition (ADOS-2)

The ADOS-2 [37] was administered by a research-reliable examiner, it includes standardized activities and ‘presses’ intended to elicit communication, social interaction, imaginative use of play materials, and repetitive behavior. The Toddler module was administered at the 24-month assessment, and Social Affect (SA), Restricted and Repetitive Behavior (RRB), and Total algorithm scores were derived. Cronbach’s alpha was .92 for the SA score and .61 for the RRB score (the lower alpha for RRB was likely due to the high number of ‘0’ and ‘1’ scores (26.15% and 23.08%, respectively).

### Statistical analysis

Analyses were run in Statistical Package for the Social Sciences (version 24, IBM). First, two multi-level repeated measures ANOVAs were run to assess mean affect and gaze separately during baseline phase, with age (12 months, 18 months) and baseline phase (baseline 1, baseline 2, baseline 3) as the embedded repeated factors, and enrollment group (LL, IL) and sex (boy, girl) as the independent between-group variables. Second, we calculated affect scores by subtracting the affect score during baseline phase 1 (before being exposed to Emotion-Evoking (EE) Task) from each phase of the EE Task to derive an affect change score for each task. We did not calculate a change score for the gaze scores. We then ran two multi-level repeated measures ANOVAs to assess mean affect and gaze separately during the phases of the EE Task, with age (12 months, 18 months) and phase (bubbles, toy play, toy removal, mask 1, mask 2, hair brushing, face washing) as the embedded repeated factors, and enrollment group (LL, IL) and sex (boy, girl) as the independent between group variables. We also completed exploratory analyses on the congruence and incongruence of the emotion expressed using a repeated measures ANOVA, with phases of the EE Task (bubbles, toy play, toy removal, mask 1, mask 2, hair brushing, face washing), age (12 months, 18 months), and evoked emotion (positive, negative, neutral) as the embedded repeated factors, and enrollment group (LL, IL) and sex (boy, girl) as the independent between-group variables. Third, we used Pearson’s *r* correlations to examine the concurrent (IBQ-R and EE Task at 12 and 18 months) associations between different measures of ER. Finally, multiple linear regressions were used to examine the utility of baseline, EE Task, and parent-reported measures for predicting later ASD symptoms (ADOS-2 Total score).

## Results

### Participant characteristics

As displayed in Table 1, data from 21 LL (14 boys and 7 girls) and 60 IL (34 boys and 26 girls) children were included in this study. There were no differences between the groups for sex, race/ethnicity, parental marital status, household income, or age for assessments at 12, 18, or 24 months (all *p*s > .05).

**Table 1:** Participant characteristics by enrollment group

Variable	Reduced likelihood (RL) Mean (SD)	Increased likelihood (IL) mean (SD)	Statistics
Age at 12-mo visit, in months	12.43 (.39)	12.38 (.61)	*t* = .37, *p* = .71
Age at 18-mo visit, in months	18.30 (.48)	18.56 (.83)	*t* = .34, *p* = .25
Age at 24-mo visit, in months	24.18 (.25)	24.77 (1.48)	*t* = .78, *p* = .44
Sex (*n* boys:girls)	14:7	34:26	*X*^*2*^ = .64, *p* = .42
Ethnicity	80% Caucasian	62% Caucasian	*X*^*2*^ = 6.28, *p* = .62
	15% Mixed	12% Mixed	
	0% Middle Eastern	7% Middle Eastern	
	0% Black	5% Black	
	0% Aboriginal	3% Aboriginal	
	5% Filipino	2% Filipino	
	0% other	9% other	
Marital status	85% married	73% married	*X*^*2*^ = 4.28, *p* = .23
	5% common law	14% common law	
	10% separated	4% separated	
	0% never lived together	9% never lived together	
Household income	5% less than $40,000	7% less than $40,000	*X*^*2*^ = 17.63, *p* = .06
	15% $40,001 – $80,000	31% $40,001 – $80,000	
	45% $80,001 – $125,000	16% $80,001 – $125,000	
	25% $125,001 – $200,000	27% $125,001 – $200,000	
	10% $200,001 and higher	5% $200,001 and higher	
	0% not given	14% not given	

### Preliminary analyses

#### Developmental age equivalents at 12 months

Group differences were explored between the children who were identified as ‘at risk’ for ASD based on ADOS-2 scores (score ≥ 8; *n *= 10). One-way ANOVAs on age equivalencies for the Mullen subscales (except Gross Motor) resulted in significant effects for the Visual Reception (*F*(2,76) = 5.86, *p* = .004) and Fine Motor (*F*(2,68) = 4.81, *p* = .01) subscales at 12 months of age. Post hoc analyses revealed that for both the Visual Reception and Fine Motor subscales, the children identified as ‘at-risk’ for ASD in the IL group had lower age equivalences compared to children in the IL group without an ASD classification, as shown in Table 2.

**Table 2:** Mean and standard deviations for age equivalencies (in months) on the Mullen

	12 months				18 months			
	LL (*a*)	IL + non ASD (*b*)	IL + ASD (*c*)	Post hoc (*p* < .004)	LL (*a*)	IL + non ASD (*b*)	IL + ASD (*c*)	Post Hoc (*p* < .03)
Visual reception	12.57 (1.47)	13.10 (1.55)	11.30 (1.64)	*c* < *b*	18.10 (2.76)	19.08 (3.09)	13.89 (4.46)	*a*, *b* > *c*
Fine motor	13.45 (1.54)	13.95 (2.38)	11.50 (0.93)	*c* < *b*	19.72 (1.93)	19.97 (2.16)	15.14 (3.72)	*a*, *b* > *c*
Receptive language	11.70 (1.66)	12.39 (2.16)	10.63 (2.45)	na	16.90 (3.89)	18.03 (4.54)	11.63 (4.31)	*a*, *b* > *c*
Expressive language	11.71 (2.37)	11.90 (2.41)	11.11 (2.67)	na	16.71 (2.45)	16.51 (2.49)	11.89 (3.14)	*a*, *b* > *c*

#### Developmental age equivalents at 18 months

Group differences were explored between the children who were identified as ‘at risk’ for ASD based on the ADOS-2 (score ≥ 8; *n* = 10). One-way ANOVAs on age equivalencies for the subscales (except Gross Motor) resulted in significant effects for the Visual Reception (*F*(2,75) = 10.11, *p* < .001), Fine Motor (*F*(2,60) = 13.26, *p* < .001), Receptive Language (*F*(2,60) = 7.16, *p* = .002), and Expressive Language (*F*(2,74) = 13.36, *p* < .001) subscales at 18 months of age. Post hoc analyses revealed that for all subscales, children ‘at risk’ for ASD in the IL group had lower age equivalences than children in the IL group without an ASD classification and children in the LL group, who did not differ.

### IBQ-R associations between 12 and 18 months

Correlations between subscales on the IBQ-R at 12 and 18 months were all statistically significant; with the lowest *r* value for high pleasure (*r* = .40, *p* = .002) and the highest *r* value for cuddliness (*r* = .71, *p* < .001). Associations between other subscales are in the Additional file 1.

### EE task associations between 12 and 18 months

#### Baseline associations between 12 and 18 months

Correlations between baseline phases were all significant for gaze [baseline phase 1 (*r* = .51, *p* < .001); phase 2 (*r* = .41, *p* < .001); phase 3(*r* = .26, *p* = .021)]. For affect, only correlations between baseline phase 3 were significant [baseline phase 1 (*r* = .17 *p* = .13); phase 2 (*r* = .19, *p* = .09); phase 3 (*r* = .26, *p* = .024)].

#### EE task associations between 12 and 18 months

Four of the seven phases for the EE Task had significant correlations between 12 and 18 months for gaze [toy play (*r* = .33, *p* = .003); toy removal (*r* = .28, *p* = .012); mask 1 (*r* = .40, *p* < .001); and mask 2 (*r* = .28, *p* = .012); not for bubbles (*r* = .02, *p* = .88); hair brushing (*r* = .05, *p* = .64); or face washing (*r* = .01, *p* = .92)]. For affect, three of the seven tasks had significant correlations [toy removal (*r* = .22, *p* = .05); mask 2 (*r* = .22, *p* = .048);and face washing (*r* = .31, *p* = .006), but not bubbles (*r* = .13, *p* = .23); toy play (*r* = .14, *p* = .21); mask 1 (*r* = .01, *p* = .88); or hair brushing (*r* = .08, *p* = .48)].

### Mean affect

#### Baseline phases

A multi-level repeated measures ANOVA found a significant effect for sex (*F*(1,70) = 4.50, *p* = .038), baseline phase (*F*(2,140) = 8.36, *p* < .001), age x group (*F*(1,70) = 4.99, *p* = .029), age x sex (*F*(1,70) = 11.64, *p* = .001), and age x group x sex (*F*(1,70) = 9.24, *p* = .003) effects. No other effects or interactions yielded significant differences.

Post hoc exploration of the sex effect using Bonferroni correction showed that girls displayed higher mean negative affect (mean ± SD = − .12 ± .25) compared to boys (mean ± SD = − .005 ± .23; *t*(72) = 2.13, *p* = .038; *d* = .39). Post hoc exploration of the baseline phase effect using Bonferroni correction showed that participants displayed higher mean negative affect during baseline phase 2 (mean ± SD = − .06 ± .36; *t*(294) = 2.52, *p* = .016; *d* = .20) and baseline phase 3 (mean ± SD = − .13 ± .47; *t*(294) = 3.55, *p* = .001; *d* = .38) compared to baseline phase 1 (mean ± SD = .002 ± .39).

Follow-up analyses of the age x group interaction showed that the LL group displayed lower mean negative affect at 18 months (mean ± SD =− .15 ± .29) compared to 12 months (mean ± SD =.02 ± .22; *t*(36) = 3.22, *p* = .018; *d* = .43); whereas there were no differences in mean affect for the IL group at 12 (mean ± SD = − .06 ± .28) or 18 months (mean ± SD = − .07 ± .22; *t*(108) = .33, *p* = .81, *d* = .02). Post hoc exploration of the age × group × sex interaction did not result in any significant relations when *p* values were adjusted using Bonferroni correction.

#### Phases of EE task (using affect change scores)

A multi-level repeated measures ANOVA found a significant effect for EE Task phase (*F*(6,420) = 16.72, *p* < .001), EE Task phase x group (*F*(6,420) = 2.73, *p* = .013), and EE Task phase x age (*F*(6,420) = 2.32, *p* = .033). No other effects or interactions were significant.

The phases of the EE Task produced the anticipated affect results for affect, with bubbles (mean ± SD = .34 ± .69) and toy play (mean ± SD = .10 ± .58) producing more positive mean affect and toy removal (mean ± SD = − .15 ± .58), mask 1 (mean ± SD = .001 ± .58), mask 2 (mean ± SD = − .06 ± .78), hair brushing (mean ± SD = − .11 ± .72), and face washing (mean ± SD = − .28 ± .88) phases producing more negative mean affect, which generally peaked at the last successive negative phase. The affective differences were confirmed with planned comparisons, showing that the bubbles phase was responded to more positively than any other phase (all *t*(146)’s > 4.35, all *p*’s < .001), and the response to the toy play phase was more positive than to the hair brushing (*t*(146) = 3.16, *p* = .002) or face washing phases (*t*(146) = 4.66, *p* < .001). For the negative phases, toy removal was more negative than mask 1 (*t*(146) = − 2.61, *p* = .01); mask 1was less negative than face washing (*t*(146) = 3.66, *p* = .001) and hair brushing (*t*(146) = 2.09, *p* = .04); and face washing was more negative than mask 2 (*t*(146) = − 2.91, *p* = .005) and hair brushing (*t*(146) = − 2.55, *p* = .01).

Planned comparisons on the EE Task phase x group showed that IL infants displayed higher rates of negative affect compared to the LL group during the hair brushing (*t*(146) = 4.72, *p* < .05; *d* = .49) and face washing phases (*t*(146) = 6.01, *p* < .05; *d* = .62).

Planned comparisons on the EE Task phase × age showed that bubbles elicited more positive affect at 18 months compared to 12 months (*t*(146) = 3.84, *p* < .05; *d* = .38). No other comparisons were significant.

#### Exploratory analyses

Statistical comparisons of the presence of evoked positive, negative, and neutral affect during each phase of the EE Task, as well as incongruent responses (e.g., negative affect during positive task) are included in the Additional file 1. Briefly, for evoked emotion, the IL group displayed more negative affect than the LL group (*t*(138) = 3.10, *p* = .016; *d* = .61) throughout the EE Task, with no group difference between positive (*t*(138) = − .45, *p* = .24; *d* = .28) or neutral (*t*(138) = − 2.14, *p* = .18; *d* = .33) expressions of affect. For incongruent responding, similar responses are seen for both groups, except for the hair brushing and face washing phases, in which the IL group had fewer displays of positive affect.

### On-task gaze

#### Baseline phases

A multi-level repeated measures ANOVA found a significant effect for age (*F*(1,75) = 8.89, *p* = .004) and baseline phase (*F*(2,150) = 6.39, *p* = .002). No other effects or interactions were significant.

Follow-up exploration of the age effect showed that participants spent more time looking at the computer screen at 18 months (mean ± SD = 72.13 ± 22.66%) compared to 12 months (mean ± SD = 63.38 ± 26.66%; *t*(156) = 2.99, *p* = .004; *d* = .31).

Follow-up exploration of the baseline phase effect showed that participants spent more time looking at the computer screen during baseline phase 1 (mean ± SD = 71.63 ± 30.67%) compared to baseline phase 2 (mean ± SD = 65.71 ± 32.68%; *t*(314) = 3.22, *p* = .002; *d* = .23) and baseline phase 3 (mean ± SD = 65.92 ± 34.55%; *t*(314) = 2.80, *p* = .006; *d* = .22), which did not differ (*t*(314) = .12, *p* = .91; *d* = .007).

#### Phases of EE task

A multi-level repeated measures ANOVA found significant effects for group (*F*(1,71) = 8.10, *p* = .006), EE Task phase (*F*(6,426) = 440.41, *p* < .001), and group x EE Task phase (*F*(6,420) = 2.30, *p* = .034). No other effects or interactions were significant. The main effect of group showed that the LL group spent more time looking at the task object (mean ± SD = 60.94 ± 6.91%) than did the IL group (mean ± SD = 55.51 ± 6.77%; *t*(72) = 2.85, *p* = .006; *d* = .48).

Examination of the main effect for EE Task phase revealed that the bubbles (mean ± SD = 88.27 ± 11.62%), toy play (mean ± SD = 84.28 ± 15.01%), mask 1 (mean ± SD = 87.61 ± 18.74%), and mask 2 (mean ± SD = 78.32 ± 19.03%) phases had the highest durations of on-task object gaze, and the phases of toy removal (mean ± SD = 53.16 ± 19.30%), hair brushing (mean ± SD = 12.95 ± 17.75%), and face washing (mean ± SD = 3.01 ± 7.03%) had lower amounts of on-task object gaze. The gaze differences were confirmed by planned comparisons, showing that all phases differed from each other (all *t*(146)’s > 2.10, all *p*’s < .03), except for mask 1 compared to bubbles (t(146) = .27, *p* = .77) and toy play (t(146) = 1.22, *p* = .21).

Planned comparisons of the EE Task x group interaction effect found that children in the LL group spent more time looking at the task object during the phases of toy removal (*t*(72) = 3.94, *p* = .05; *d* = .32), mask 1 (*t*(72) = 4.94, *p*= .02; *d* = .40), and mask 2 (*t*(72) = 5.51, *p =* .01; *d* = .45) compared to the IL group. The groups did not differ on on-task gaze for the phases of bubbles, toy play, face washing, or hair brushing.

### Concurrent association with parent-reported temperament

To test the validity of our EE Task, we ran correlations between affect and gaze scores during the EE Task and subscale scores on the IBQ-R at the 12-month and 18-month time-points. Because of the many statistical comparisons, we corrected the *p* value by number of Baseline and EE Task activities (*n* = 10), flagging only those correlations with *p* < .005 as statistically significant. Results are presented below for all participants combined, followed by the IL group alone and the LL group alone.

### All participants

Overall, at 12 or 18 months, affect and on-task gaze scores for the EE Task were concurrently associated with 3 of 14 IBQ-R scales.

#### 12 months

Correlations between IBQ-R subscales and EE Tasks for all participants at 12 months are shown in Table 3. There were no significant associations with a *p* value of < .005 for affect or gaze.

**Table 3:** Concurrent associations between subscales on the IBQ-R and phases on the EE task at 12 months

	Baseline 1	Baseline 2	Baseline 3	Bubbles	Toy play	Toy removal	Mask 1	Mask 2	Hair brushing	Face washing
*Affect*										
Activity level	.09	− .10	.05	− .05	− .07	− .30	− .05	− .10	.06	.06
Distress to limitations	.002	.007	− .009	− .06	− .05	− .14	.04	.13	.16	.21
Fear	.14	.15	− .02	.09	.03	.02	.07	.14	− .03	.01
Duration of orienting	.02	− .16	− .07	.14	.004	.09	− .02	.09	− .16	.01
Smile and laughter	.02	− .05	.001	.18	.04	.09	.14	.17	− .02	.19
High pleasure	− .13	− .12	− .04	.18	.07	.20	.15	.04	.16	.27
Low pleasure	− .02	− .06	− .07	− .08	0	.27	− .02	.07	− .09	− .01
Soothability	− .06	.04	.04	.20	.17	.14	− .10	.003	.14	.15
Falling reactivity	.10	.19	.08	.14	.29	.14	.07	.12	− .20	− .06
Cuddliness	− .10	− .09	.02	.08	.09	.23	.18	.02	.10	− .04
Perceptual sensitivity	.09	− .02	− .006	− .11	− .006	.03	− .06	.08	− .18	− .05
Sadness	− .009	.06	− .08	− .02	.07	− .18	.10	.13	− .01	.17
Approach	.01	− .06	.12	.04	.05	.18	.23	.22	.03	.22
Vocal reactivity	− .11	− .09	− .09	.22	0	.20	.26	.18	− .02	.11
*Gaze*										
Activity level	− .09	− .04	.01	.15	.08	.07	− .02	.07	.24	.11
Distress to limitations	− .05	− .03	− .14	.02	.04	.08	.02	.08	.14	.09
Fear	− .007	− .03	− .06	− .05	.03	.08	.01	.12	.15	− .008
Duration of orienting	− .06	− .05	− .09	− .01	.013	.05	− .04	.06	.04	.11
Smile and laughter	− .17	− .19	− .16	− .13	− .08	− .05	− .05	− .002	.15	− .08
High pleasure	− .04	− .16	− .02	− .01	− .08	.03	.02	− .06	.06	− .20
Low pleasure	.03	.06	.08	.009	.04	− .05	.15	.05	.01	.02
Soothability	− .25	− .30	− .28	− .13	− .06	.01	0	− .14	− .07	− .12
Falling reactivity	− .19	− .24	− .29	− .02	− .15	− .14	− .14	− .13	− .07	− .09
Cuddliness	− .09	− .13	− .05	− .23	− .07	− .09	− .09	− .09	.15	− .17
Perceptual sensitivity	− .17	− .03	− .14	− .02	.06	− .01	− .06	.10	− .02	− .16
Sadness	.01	− .01	.007	.04	.05	.21	.007	.12	− .02	− .09
Approach	.007	− .10	− .01	− .03	− .09	.07	.02	− .02	.07	− .03
Vocal reactivity	− .05	− .08	.003	.04	− .05	.10	.17	.03	.13	.10

#### 18 months

Correlations between IBQ-R subscales and EE Task for all participants at 18 months are shown in Table 4. Three associations for affect and one for gaze were significant with a *p* value of < .005. Higher negative affect during the hair brushing phase was associated with endorsement on the IBQ-R of higher rates of fussiness and distress when in a confined space, during caretaking activities, or inability to do a preferred action (distress to limitations), as well as displaying low mood and activity (sadness). Similarly, higher negative affect during the mask 2 phase was also associated with ratings of lower mood and activity (sadness) on the IBQ-R. Decreased on-task gaze during baseline phase 1 was associated with ratings indicating greater detection of slight, low intensity stimuli in the child’s environment (perceptual sensitivity) on the IBQ-R.

**Table 4:** Concurrent associations between subscales on the IBQ-R and phases on the EE task at 18 months

	Baseline 1	Baseline 2	Baseline 3	Bubbles	Toy play	Toy removal	Mask 1	Mask 2	Hair brushing	Face washing
*Affect*										
Activity level	− .08	.03	− .06	.33	.16	.03	.19	.06	− .15	.001
Distress to limitations	.04	.05	− .17	.12	− .06	− .09	− .23	.32	− .38*	− .06
Fear	− .012	.15	.02	.16	.08	0	− .21	− .26	− .23	− .05
Duration of orienting	.07	− .005	− .08	.24	.06	− .22	.009	− .10	− .19	− .02
Smile and laughter	− .06	− .12	.06	− .10	.12	.12	− .04	− .002	.09	.03
High pleasure	.05	− .05	.22	− .12	− .03	− .11	− .05	− .06	.03	− .11
Low pleasure	− .21	.05	− .07	.09	.10	.08	.03	− .17	− .05	.23
Soothability	− .006	.011	.13	− .26	.03	.16	− .06	− .05	.12	.08
Falling reactivity	− .10	.08	.18	− .14	.03	.18	.05	.02	.17	− .09
Cuddliness	− .16	− .03	.12	− .19	.14	.18	− .03	.05	.11	.16
Perceptual sensitivity	− .008	.11	− .04	− .13	− .06	.06	− .09	− .02	− .14	.11
Sadness	.02	.008	− .18	.08	− .08	− .31	− .14	− .43*	− .37*	.02
Approach	.09	.05	.03	− .002	− .05	.02	− .10	− .20	− .14	− .01
Vocal reactivity	− .01	− .17	− .01	− .15	.04	.009	− .18	− .19	.03	0
*Gaze*										
Activity level	− .03	− .03	− .04	.13	− .02	.02	.02	.04	.11	.20
Distress to limitations	− .05	.01	− .07	.04	.13	.07	− .02	− .24	− .11	.05
Fear	− .03	.02	.002	.11	.10	− .13	− .13	− .08	.002	.02
Duration of orienting	.006	.10	.02	.25	− .09	− .26	.14	− .14	.01	.31
Smile and laughter	− .23	− .17	− .11	.003	.035	.05	− .10	− .02	− .11	.08
High pleasure	.03	− .01	.08	− .13	.04	.06	.02	.02	.03	.07
Low pleasure	− .18	− .11	− .12	.13	− .11	− .17	− .09	− .15	− .13	.12
Soothability	− .16	− .15	.003	− .22	− .09	.06	− .12	− .006	.09	− .05
Falling reactivity	− .07	− .10	− .07	− .23	− .10	− .05	− .20	− .02	.10	.07
Cuddliness	− .09	− .03	.07	− .18	− .16	.13	− .06	.16	− .08	.08
Perceptual sensitivity	− .47*	− .19	− .19	− .05	− .05	− .13	− .03	− .11	− .10	.09
Sadness	.01	.06	.03	.02	.26	− .05	− .04	− .28	− .09	.03
Approach	− .19	− .19	− .06	− .04	.07	− .05	.07	− .07	− .17	.02
Vocal reactivity	− .19	− .10	− .10	− .08	− .11	− .04	− .18	− .15	− .15	.07

Bolded items indicate significant results following multiple corrections

*p < .005

### IL group

Scores on the EE Task were associated with 7 of 14 scales on the IBQ-R.

#### 12 months

No significant associations were seen between affect or gaze ratings during baseline phases 1, 2, or 3 and IBQ-R subscales. For phases of the EE Task, there were no associations with gaze but there were significant associations with affect. Mask 1 was positively associated with high pleasure (*r* = .42, *p* = .003), approach (*r* = .41, *p* = .004), and vocal reactivity (*r* = .51, *p* < .001). These relationships suggest that children who displayed higher levels of positive affect during Mask 1 were also rated as showing increased levels of pleasure to situations with high stimuli of novel and complex intensity (high pleasure), increased approach and anticipation of pleasurable activities (approach), and engagement in high rates of vocalization throughout the day (vocal reactivity).

#### 18 months

During baseline phase 1, affect was negatively associated with low pleasure (*r* = − .49, *p* = .001) and gaze was negatively associated with perceptual sensitivity (*r* = − .43, *p* = .004). These relations suggest that children who displayed increased negative affect during baseline phase 1 were endorsed on the IBQ-R as showing higher interest in situations with reduced amounts of stimuli of novel and complex intensity (low pleasure). Similarly, children who spent less time looking at the monitor were rated as showing increased interest in low intensity stimuli in their environment (perceptual sensitivity).

For phases of the EE Task, there were no associations with gaze, but there were significant associations for affect. Mask 2 was negatively associated with endorsement of sadness (*r* = − .50, *p* < .001) and hair brushing was negatively associated with distress to limitations (*r* = − .42, *p* = .005). These relations suggest that increased negative affect during masks 2 was associated with parental IBQ-R ratings of increased levels of low mood and activity (sadness). Similarly, higher negative affect during the hair brushing phase was associated with endorsement of higher rates of fussiness and distress when in a confined space, during caretaking activities, or inability to do a preferred action (distress to limitations).

### LL group

Overall, scores on the EE Task were associated with 2 of 14 scales on the IBQ-R.

#### 12 months

There were no significant associations for affect or gaze and IBQ-R subscales during the phases of baseline. During the EE Task, affect during the mask 2 phase was associated with IBQ-R falling reactivity/ recovery rate (*r* = .68, *p* = .002) and gaze during face washing phase was associated with endorsement of cuddliness (*r* = − .76, *p* < .001). These relations suggest that increased negative affect during masks 2 was associated with parental ratings of prolonged recovery from peak distress or excitement (falling reactivity). Similarly, higher on-task gaze during face washing was related to ratings of increased expression of enjoyment while being held by a caregiver (cuddliness).

#### 18 months

There were no associations for affect or gaze during the baseline phases or phases of the EE Task and IBQ-R subscales.

### Predictive association with ASD symptoms

Hierarchical linear regressions were performed with Total ADOS-2 score at 24 months as the dependent variable and baseline phases, EE task phases, and IBQ-R subscales at 12 and 18 months as separate predictor variables. All regression models included enrollment group (IL, RL) as an independent predictor in model 2 and age equivalencies on the receptive and expressive subscales of the Mullen as independent predictors in model 3.

#### Predictors

We first ran linear regressions with our participant characteristics (enrollment group, sex, receptive language age equivalence, expressive language age equivalence) to determine if they predicted ADOS Total Severity Scores alone. Enrollment group (*R*^2^ = .05; *F*(1,63) = 3.62, *p* = .06) and sex (*R*^2^ = .04; *F*(1,63) = 2.88, *p* = .09) did not predict ADOS Total Severity Scores. Similarly, receptive and expressive age equivalencies were not predictive at 12 months (*R*^2^ = .04; *F*(2,52) = .93, *p* = .40), but were predictive at 18 months (*R*^2^ = .31; *F*(2,47) = 10.58, *p* < .001). Because we were interested in exploring differences between the IL and LL groups, and the regression trended towards significance, we included enrollment group as a predictor in the models, in addition to age equivalencies at 12 and 18 months.

#### Gaze and affect at 12 months

##### Baseline

For affect, all three models were not significant [Model 1: (*R*^2^ = .03; *F*(3,57) = .51, *p* = .68; Model 2: (*R*^2^ = .08; *F*(4,56) = 1.27, *p* = .29); Model 3 (*R*^2^ = .18; *F*(6,46) = 1.69, *p* = .14)]. For gaze, Models 1 (*R*^2^ = .13; *F*(2,59) = 2.87, *p* = .04) and 2 (*R*^2^ = .16; *F*(4,58) = 2.72, *p* = .038) were significant, whereas Model 3 was not (*R*^2^ = .13; *F*(6,46) = 1.11, *p* = .37). As shown in Table 5, examination of the coefficients identified that baseline phase 3 was a significant predictor for Model 1 (*β* = − .11, *p* = .005) and Model 2 (*β* = − .10, *p* = .009).

**Table 5:** Predictive relationships between 12-month affect and gaze and 24-month ADOS total severity score

Variable	Model 1		Model 2		Model 3	
	*B*	*β*	*B*	*β*	*B*	*β*
*Affect: baseline*						
Constant	6.21		6.87		13.21	
Baseline 1	.57	.04	. .40	.03	1.03	.07
Baseline 2	− 2.33	− .17	− 2.96	− .21	− 4.28	− .29
Baseline 3	2.03	.19	2.43	.22	2.68	.24
Enrollment group	–	–	− 2.75	− .24	− 3.57	− .31
Receptive language 12 months	–	–	–	–	− .62	− .28
Expressive language 12 months	–	–	–	–	.11	.06
*R*^*2*^	.03		.09		.18	
*F (p)*	.51 (.68)		1.27 (.29)		1.69 (.14)	
*Gaze: baseline*						
Constant	6.21**		6.72**		11.74	
Baseline 1	.04	.15	.04	.16	− .05	− .22
Baseline 2	.07	.30	.06	.26	.06	.25
Baseline 3	− .11**	− .53	− .10**	− .50	− .20	− .10
Enrollment group	–	–	− 2.27	− .18	− 3.05	− .25
Receptive language 12 months	**–**	–	–	–	− .51	.22
Expressive language 12 Months	–	–	–	–	.20	.09
*R*^*2*^	.13		.16		.13	
*F (p)*	2.87 (.04)*		2.72 (.04)*		1.11 (.37)	
*Affect: EE task*						
Constant	5.61		6.00		12.51	
Bubbles	1.06	.08	1.09	.08	.57	.04
Toy play	− 1.41	− .08	− 1.44	− .09	− .59	− .03
Toy removal	− 1.13	− .08	− .65	− .04	.006	0
Mask 1	4.05	.25	3.84	.24	3.95	.24
Mask 2	− 2.12	− .20	− 2.26	− .22	− 2.75	− .26
Hair brushing	− 3.22	− .26	− 2.78	− .22	− 4.77	− .30
Face washing	.18	.02	.15	.02	− .58	− .05
Enrollment group	-	-	− 1.42	− .11	− 1.71	− .13
Receptive language	-	-	-	-	− .57	− .24
Expressive language	-	-	-	-	− .001	− .001
*R*^*2*^	.13		.14		.29	
*F (p)*	1.11 (.37)		1.04 (.42)		1.69 (.12)	
*Gaze: EE task*						
Constant	13.24***		12.68***		13.24	
Bubbles	− .002	− .006	− .01	− .04	.03	.12
Toy play	.07	.24	.09	.31	.05	.19
Toy removal	− .05	− .18	− .05	− .19	− .10	− .39
Mask 1	− .07	− .27	− .06	− .25	− .05	− .19
Mask 2	− .06	− .25	− .06	− .24	− .03	− .12
Hair brushing	.02	.09	.01	.05	.01	.05
Face washing	− .06	− .12	− .04	− .09	− .09	− .21
Enrollment group	–	–	− 2.15	− .16	− 2.07	− .17
Receptive language	–	–	–	–	− .61	− .26
Expressive language	–	–	–	–	.42	.20
*R*^*2*^	.25		.27		.27	
*F (p)*	2.52 (.026)*		2.41 (.027)*		1.48 (.18)	

Bolded items indicate significant results following multiple corrections

*p < .05, **p < .01, ***p < .001 for co-efficient; ‘–’ not included in model

##### EE task

For affect, all three models were not significant [Model 1: (*R*^2^ = .13; *F*(7,53) = 1.11, *p* = .37; Model 2: (*R*^2^ = .14; *F*(8,52) = 1.04, *p* = .42); Model 3 (*R*^2^ = .29; *F*(10,41) = 1.69, *p* = .12)]. For gaze, Models 1 (*R*^2^ = .25; *F*(7,52 = 2.51, *p* = .026) and 2 (*R*^2^ = .27; *F*(8,51) = 2.41, *p* = .027) were significant, whereas Model 3 was not (*R*^2^ = .28; *F*(10,39) = 1.48, *p* = .18). As shown in Table 5, examination of the coefficients did not identify any significant effects for gaze in Table 6, examination of the coefficients identified the toy removal (*β* = − .08, *p* = .026) , mask 1 (*β* = − .12, *p* = .011), and hair brushing (*β* = .09, *p* = .048) phases as significant predictors for Model 1; the mask 1 (*β* = − .11, *p* = .018) and hair brushing (*β* = .09, *p* = .037) phases as significant predictors for Model 2; and the mask 1 phase (*β* = − .14, *p* = .009) and receptive language age equivalence (*β* = − .46, *p* = .018) as significant predictors for Model 3.

**Table 6:** Predictive relationships between 18-month affect and gaze and 24-month ADOS total severity score

Variable	Model 1		Model 2		Model 3	
	*B*	*β*	*B*	*β*	*B*	*β*
*Affect: baseline*						
Constant	7.09		7.99***		22.29***	
Baseline 1	− 2.39	− .13	− 1.83	− .10	− 2.99	− .14
Baseline 2	3.69	.16	3.67	.16	3.06	.14
Baseline 3	2.86	.17	3.22	.19	4.28	.25
Enrollment group	–	–	− 3.83*	− .28	− 3.86*	− .30
Receptive language	–	–	**–**	–	− .52**	− .42
Expressive language	–	–	**–**	–	− .32	.17
*R*^*2*^	.08		.15		.49	
*F (p)*	1.60 (.20)		2.59 (.046)*		6.71(<.001)***	
*Gaze: baseline*						
Constant	2.64		3.63		19.26***	
Baseline 1	.07	.22	.06	.20	.003	.009
Baseline 2	− .02	− .06	− .005	− .02	− .004	− .02
Baseline 3	.003	.01	− .008	− .04	.05	.20
Enrollment group	–	–	− 2.97	− .21	− 3.12	− .24
Receptive language	–	–	–	–	− .42*	− .34
Expressive language	–	–	–	–	− .51	− .27
*R*^*2*^	.04		.08		.40	
*F (p)*	.75 (.53)		1.30 (.28)		4.74 (.001)***	
*Affect: EE task*						
Constant	5.84***		6.34***		21.01***	
Bubbles	− .28	− .02	.61	.04	− 1.56	− .09
Toy play	− .37	− .02	− .45	− .03	− .60	− .04
Toy removal	2.76	.18	2.35	.15	3.40	.21
Mask 1	− 1.13	− .09	− .74	− .06	− 2.30	− .14
Mask 2	3.99*	.40	3.66*	.36	1.76	.13
Hair brushing	.58	.05	1.54	.14	.29	.20
Face washing	− 5.28***	− .48	− 5.34***	− .49	− 4.38*	− .29
Enrollment group	**–**	–	− 3.39*	− .25	− 1.60	− .12
Receptive language	**–**	–	**–**	–	− .27	− .22
Expressive language	**–**	–	–	–	− .60	− .32
*R*^*2*^	.26		.31		.49	
*F (p)*	2.75 (.016)*		3.09 (.006)**	3.58 (.002)**		
*Gaze: EE task*						
Constant	11.28		10.43		21.48**	
Bubbles	.07	.15	.07	.16	.07	.16
Toy play	− .006	− .02	− .01	− .03	.002	.006
Toy removal	− .08*	− .31	− .07	− .27	− .05	− .17
Mask 1	− .12*	− .43	− .11*	− .40	− .14**	− .47
Mask 2	.04	.17	.04	.17	.04	.14
Hair brushing	.09*	.27	.09*	.29	− .02	− .05
Face washing	− .02	− .10	− .09	− .04	.15	.04
Enrollment group	–	–	− 1.76	− .13	− 2.28	− .17
Receptive language	–	–	–	–	− .46*	− .37
Expressive language	–	–	–	–	− .11	− .06
*R*^*2*^	.25		.27		.53	
*F (p)*	2.63 (.021)*		2.41 (.027)*		4.08 (.001)***	

Bolded items indicate significant results following multiple corrections

^*^ *p* < .05, ** *p* < .01, *** *p* < .001 for co-efficient; ‘-‘ not included in model

#### Gaze and affect at 18 months

##### Baseline

For affect, Model 2 (*R*^2^ = .15; *F*(4,558 = 2.59, *p* = .046) and Model 3 (*R*^2^ = .49; *F*(6,42) = 6.71, *p* < .001) are significant, whereas Model 1 was not (*R*^2^ = .08; *F*(3,59) = 1.60, *p* = .20). As shown in Table 6, examination of the coefficients identified that enrollment group (*β* = − 3.83, *p* = .026) was a significant predictor for Model 2 and enrollment group (*β* = − 3.86, *p* = .014) and receptive language age equivalence (*β* = − .52, *p* = .006) were significant predictors in Model 3.

For gaze, Models 1 (*R*^2^ = .04; *F*(3,60) =.75, *p* = .53) and 2 (*R*^2^ = .08; *F*(4,59) = 1.30, *p* = .28) were not significant, whereas Model 3 was significant (*R*^2^ = .40; *F*(6,42) = 4.74, *p* = .001). As shown in Table 6, examination of coefficients identified that receptive language age equivalence was a significant predictor for Model 3 (*β* = − .42, *p* = .033).

##### EE task

For affect, all three models were significant [Model 1: (*R*^2^ = .26; *F*(7,55) = 2.75, *p* = .016); Model 2: (*R*^2^ = .31; *F*(8,54) = 3.09, *p* = .006); Model 3 (*R*^2^ = .49; *F*(10,37) = 3.38, *p* = .002)]. As shown in Table 6, examination of the coefficients identified the mask 2 (*β* = 3.99, *p* = .038) and face washing (*β* = − 5.28, *p* = .001) phases as significant predictors for Model 1; mask 2 (*β* = 3.66, *p* = .05) and face washing (*β* = − 5.34, *p* = .001) phases, as well as enrollment group (*β* = − 3.39, *p* = .043) as significant predictors for Model 2; and the face washing phase (*β* = − 4.38, *p* = .036) as a significant predictor for Model 3.

For gaze, all three models were significant [Model 1: (*R*^2^ = .26; *F*(7,55) = 2.75, *p* = .016); Model 2: (*R*^2^ = .31; *F*(8,54) = 3.09, *p* = .006); Model 3 (*R*^2^ = .40; *F*(10,36) = 4.08, *p* = .001)]. As shown in Table 6, examination of the coefficients identified the toy removal (*β* = − .08, *p* = .026) , mask 1 (*β* = − .12, *p* = .011), and hair brushing (*β* = .09, *p* = .048) phases as significant predictors for Model 1; the mask 1 (*β* = − .11, *p* = .018) and hair brushing (*β* = .09, *p* = .037) phases as significant predictors for Model 2; and the mask 1 phase (*β* = − .14, *p* = .009) and receptive language age equivalence (*β* = − .46, *p* = .018) as significant predictors for Model 3.

#### IBQ-R at 12 and 18 months

##### 12 months

As shown in Table 7, all three models were not significant [Model 1: (*R*^2^ = .13; *F*(714,41) =.44, *p* = .95; Model 2: (*R*^2^ = .14; *F*(15,40) =.45, *p* = .95); Model 3 (*R*^2^ = .25; *F*(17,31) = .60, *p* = .87)].

**Table 7:** Predictive relationships between 12- and 18-month IBQ-R and 24-month ADOS total severity score

Variable	Model 1		Model 2		Model 3	
	*B*	*β*	*B*	*β*	*B*	*β*
*12 months: IBQ-R*						
Constant	9.05		10.82		17.32	
Activity level	− .63	− .10	− .43	− .07	− .52	− .08
Distress to limitations	− .41	− .07	− .66	− .11	− .96	− .14
Fear	.26	.05	.05	01	− .39	− .07
Duration of orienting	.78	.16	.67	.13	.64	.13
Smile and laughter	1.57	.29	1.29	.24	1.58	.29
High pleasure	2.59	.28	2.45	.27	2.62	.28
Low pleasure	− 1.00	− .17	− 1.05	− .18	− .81	− .13
Soothability	− 1.46	− .18	− 1.22	− .15	− 2.79	− .32
Falling reactivity	− .57	− .09	− .65	− .10	− .58	− .08
Cuddliness	.64	.08	.58	.07	1.85	.22
Perceptual sensitivity	.54	.13	.51	.12	1.18	.25
Sadness	− .16	− .03	− .17	− .03	− .16	− .03
Approach	− 1.30	− .17	− 1.27	− .17	− 1.92	− .24
Vocal reactivity	− 1.39	− .27	− 1.03	− .20	− 1.38	− .26
Enrollment group	–	–	− 1.67	− .14	− 2.31	− .18
Receptive language	–	–	–	–	− .24	− .10
Expressive language	–	–	–	–	− .03	− .02
*R*^*2*^	.13		.14		.25	
*F (p)*	.44 (.95)		.45 (.95)		.60 (.87)	
*18 months: IBQ-R*						
Constant	14.99		15.40		30.07	
Activity Level	1.21	.19	1.39	.21	.07	.01
Distress to Limitations	− .93	− .17	− .84	− .15	− 1.08	− .20
Fear	− .48	− .10	− .75	− .15	.14	.03
Duration of orienting	.64	.13	.44	.09	.15	.03
Smile and laughter	2.95	.49	2.35	.39	1.39	.25
High pleasure	.10	.01	.77	.09	.36	.04
Low pleasure	− .02	− .003	.06	.009	.14	.02
Soothability	− .71	− .10	− .71	− .10	.02	.01
Falling reactivity	− .76	− .14	− .79	− .14	− .99	− .20
Cuddliness	1.09	.19	.97	.17	.70	.13
Perceptual sensitivity	.17	.04	.30	.07	.68	.16
Sadness	.61	.12	.50	.10	.02	.01
Approach	− 2.90	− .33	− 3.50	− .39	− 2.13	− .26
Vocal reactivity	− 1.77	− .27	− 1.11	− .17	− .95	− .16
Enrollment group	-	–	− 2.72	− .22	− 3.55	− .31
Receptive language	–	–	–	–	− .41	− .38
Expressive language	–	–	–	–	− .38	− .22
*R*^*2*^	.27		.31		.54	
*F (p)*	.92 (.55)		.98 (.50)		1.44 (.21)	

‘–’ Not included in model

##### 18 months

As shown in Table 7, all three models were not significant [Model 1: (*R*^2^ = .27; *F*(14,34) =.92, *p* = .55; Model 2: (*R*^2^ = .31; *F*(15,33) =.98, *p* = .50); Model 3 (*R*^2^ = .54; *F*(17,21) = 1.44, *p* = .21)].

## Discussion

We explored behavioral responses (affect and gaze) to emotionally salient stimuli at 12 and 18 months of age by children who were at a low or increased likelihood for a later diagnosis of ASD. Parents completed the IBQ-R temperament questionnaire at 12 and 18 months, and all children received an ADOS-2 assessment for ASD symptomatology at 24 months. There were three main results. First, the IL group showed higher rates of negative affect and spent less time looking at the task objects compared to the LL group during the Emotion-Evoking Task. Second, affect and gaze showed concurrent associations with several IBQ-R subscales for both the LL and IL groups. Third, gaze at 12 months and gaze and affect at 18 months, but not IBQ-R scores, predicted ADOS-2 scores at 24 months in the IL group. These results suggest that behavioral responses to emotionally salient stimuli may provide important information to support early detection of emerging ASD symptoms, complementing parent ratings of temperament in IL children as early as 12 months of age.

A critical consideration when assessing ER is to determine whether the tasks are producing the expected result (i.e., the putatively negative tasks elicit negative responses [55]). The tasks used in this study were adapted from the Lab-TAB [24] and were designed to probe-specific emotions. Comparisons across our tasks showed increasingly negative responses following bubbles (most positive) to face washing and hair brushing (most negative). Participants also spent more time looking at the more positive tasks (bubbles and toy play) and less time looking at the negative tasks, particularly toy removal, hair brushing, and face washing. The reduced time spent looking at on-task objects during hair brushing and face washing may also be related to the difficulty of looking at a comb/brush and face cloth during these tasks, as well as attempts to avoid (move away from) the brush and face cloth. Some children in both groups responded in ways that were incongruent with the probed emotion, for example, smiling during toy removal. Despite this individual variability, we showed that the vast majority of responses aligned with the probed emotion, which may reflect the validity of the task (positive tasks were experienced as positive, and vice versa), and the placement of a neutral task between the positive and negative tasks to allow time to recover from the previous emotionally salient stimuli [55, 58]. That our tasks appear valid is important because we chose tasks that children could experience in their day-to-day life that would be emotionally valanced (positive or negative) without being too emotionally arousing for the children (evidenced by the low means [< ± 1] for affect during negative and positive tasks).

As noted, we included three baseline periods within our testing protocol. The first allowed participants to acclimate to the testing environment and provided baseline values for affect and gaze, the second allowed an opportunity to recover to minimize carry-over from positive to negative tasks, and the third provided an opportunity to recover from stress produced by the negative tasks, per methodological recommendations [55, 58]. Although we did collect heart rate data in this study, these were not examined in the current report. We did, however, follow the protocol for testing autonomic nervous system reactivity (calculating the difference between affective responses during the emotionally salient stimuli and baseline [35]). Comparisons of affect and gaze during baseline showed no differences between the LL and IL groups. Participants (collectively) had slightly more negative affective responses and spent less time looking at the screen during baselines 2 and 3 compared to baseline 1. Evaluation of ER during baseline is important as it provides a measure of the child’s ability to regulate their emotions [2]. That our participants showed more negative affect and spent less time looking at the computer screen during successive baseline periods may be the result of (1) the EE Task, highlighting the importance of baseline periods to minimize carry-over effects and reduce cumulative stress to the child caused by emotionally challenging tasks, (2) the child becoming restless or fatigued from the EE Task, and/or (3) the child becoming bored by the baseline video, which was the same across the three baseline periods.

The LL and IL groups showed differential responding during the emotionally salient tasks, as predicted. The IL group displayed higher rates of negative affect and spent less time looking at the task objects compared to the LL group, in accordance with previous research on parent ratings of temperament in children diagnosed with ASD [7,36,57, 21]. Although there is a paucity of research on observed ER in children under 2 who are at increased likelihood of/diagnosed with ASD, a few studies have explored ER in children between ages 2 and 5 years. Jahromi et al. [34] assessed facial affect in 4-year-old children with and without ASD during two frustration tasks (toy in a locked box and unsolvable puzzle) and found no differences between the two groups. Similarly, Zantinge et al. [67] presented 5-year-old children with and without ASD with an unpredictable toy robot and recorded facial affect; again, the researchers did not find group differences. Hirschler Guttenberg et al. [30] measured affect and gaze during tasks designed to elicit fear (experimenter wears masks) and joy (child and parent play with hand puppets) in 5-year-old children with and without ASD. Although no differences were found for gaze, positive emotions were reduced and fear was increased during the fearful task in children with ASD, but only when fathers rather than mothers were present. The protocol that most closely resembled ours was carried out by Macari et al. [39]. Two-year old-children with ASD and neurotypical children participated in tasks designed to elicit anger, fear, and joy using tasks from the Lab-TAB. The researchers found that children with ASD displayed lower intensity fear, but no differences for anger or joy when compared to neurotypical peers.

Our findings of differences between the LL and IL groups may be explained by differences in methodology relative to previous studies. First, our participants were tested at younger ages [12 and 18 months vs. ~20 [39] or ~50 months [30, 34, 67]], and as such, may be more reactive because ER systems are still developing. Second, the previous studies included smaller samples and selected children with higher cognitive and language functioning [34]. Our relatively large sample of IL children was tested at two time-points, and we did not select participants based on level of cognitive or language ability. Third, we employed shorter intervals for coding affect (5 s) compared to the 10-s (or longer) intervals used by Jahromi et al. [34], Macari et al. [39], and Zantinge et al. [67], which may have allowed us to capture more nuanced changes in affect.

As predicted, the validity of our Emotion-Evoking Task relative to assessing emotion regulation was supported by concurrent relations with temperament on the parent-reported IBQ-R. Interestingly, when both the IL and LL groups were combined, significant relationships were not found at 12 months of age, but were found for both affect (mask 1, mask 2, and hair brushing) and gaze (baseline 1) at 18 months. When separated out, the IL group did show significant relationships between three subscales on the IBQ-R at 12 months and affective responses on the mask 1 phase. At 18 months, affect and gaze during baseline phase 1 (before any EE Task phase) was associated with low pleasure and perceptual sensitivity, and affective responses during mask 2 and hair brushing were associated with sadness and distress to limitation, respectively. For the LL group, affect during mask 2 was associated with recovery rate and gaze during face washing was associated with cuddliness. There were no relationships for the LL group at 18 months. These finding are important because they suggest our EE Task shows convergent validity with parent-reported temperament, specifically the affective responses during negative tasks for mask 1, mask 2, hair brushing, and face washing and gaze durations for baseline 1). These results are in line with a recent review by Sacrey et al. [55], which reviewed physiological and affective responses during emotionally salient tasks and found that the overwhelming majority of studies used negatively salient tasks to elicit responses. That there were relationships between EE Task and the IBQ-R at 18 months for the combined and IL group, but not the LL group may be due to the age parameters of the IBQ-R. The IBQ-R had suggested use for infants between 6 and 12 months of age. We included it here at 18 months both for consistency between time points for the parent-reported measure and the EE Task, but also due to variability in the developmental age of the IL group (which was confirmed by the Mullen subscales at 18 months). Nevertheless, all subscales of the IBQ-R were significantly correlational with each other at 12 and 18 months. Temperament is viewed as the biologically based disposition to express certain emotions when challenged, and with development we learn to regulate our expressed emotions with respect to our inherent disposition using a variety of ER strategies [18].

Associations between affect, gaze, and IBQ-R scales and ADOS-2 Total scores supported our prediction that affect and gaze would predict ASD symptoms at age 2. The discriminatory ability of affect and gaze was important for the IL group. Gaze at 12 months and both affect and gaze at 18 months were predictive of 24-month ADOS scores in the IL group. Differences in ER have been associated with later mental health disorders [32], as maladaptive ER strategies tax our cognitive capacity and increase autonomic arousal, resulting in long-term ER dysregulation [4, 25]. As such, our results are in accordance with studies that report a higher prevalence of emotional difficulties in children with ASD compared to neurotypical children [12] and children with intellectual disability [6]. Rates of emotional difficulties in children with ASD are reported to range from 71 to 86% [50, 61], with over 50% reporting four or more internalizing or externalizing problems [43]. Because emotional difficulties can have negative effects on a child’s academic ability and quality of life, as well as on their families [19, 66, 60], the earlier ER difficulties can be identified, the earlier interventions can be implemented. For example, the Attachment and Biobehavioral Catch-Up intervention has been shown to improve emotional dysregulation through mother-oriented strategies in emotionally dysregulated infants as young 12 months [28], although long-term effects will be important to demonstrate.

## Strengths and limitations

Our study has several strengths; we measured behavioral responses to positively and negatively valanced tasks twice prior to age 2, we included three baseline periods to minimize carry-over effects between positive and negative tasks, our effect sizes were within the medium range, and our sample of IL infant siblings was relatively large.

Limitations include first that there may be a difference in ER between IL siblings and children with non-familial ASD; as such, our results may not be generalizable to non-IL samples. Second, due in part to the age of participants, we did not identify outcomes based on clinical best estimate diagnosis (ASD versus no ASD), but rather compared LL and IL groupings and used ADOS-2 scores as an index of ASD symptoms. Third, the lesser percentages of time spent looking at the on-task object for the IL group may have impacted the affect results. That the IL group spent less time looking at the mask 1, mask 2, and toy removal phases, but did not differ in affective responding from the LL group, may suggest that the IL group was gazing away from the on-task object as a means of regulating their affective response [62]. Further examination in the different types of gaze used during the phases of the EE Task (e.g., looking at parent), is warranted. However, there is value in examining early ASD symptoms on a continuum, especially in relation to emotion regulation in siblings of children with ASD, for whom a higher prevalence of mental health difficulties is an additional concern beyond increased likelihood of ASD [31].

Future work will include comparison of affect and gaze between IL siblings stratified by ASD diagnosis at age 3. Nevertheless, the current study contributes to the growing evidence that ER difficulties are one of the earliest expressions of ASD vulnerability and manifest as early as 12 months of age. These results have the potential to inform ASD surveillance efforts as well as novel treatment strategies to interrupt pathways between emotional dysregulation and academic, behavioral, and social impairments [5,14,15,49,63,65].

## Conclusions

Our study is the first to show that children with increased familial likelihood of an ASD diagnosis have differences from children at community-level risk in directly observed behavioral responses to emotionally evocative stimuli by as young as 12 months. These findings add to the cumulative evidence that children at IL for ASD have very early ER difficulties. Observed behavioral responses in the IL sample, but not parent ratings on the IBQ-R, were associated with later ASD symptoms, highlighting the importance of directly observing behavioral responses in emotionally salient situations. The associations between increased negative affect for participants on the mask 1, mask 2, hair brushing, and face washing phases and parent endorsement of more problematic scores on the scales that measure distress or sadness when placed in a confined position, when barred from performing a desired activity, or when engaged in caretaking activities may help focus future work on ER in children with ASD to those tasks and scales that show the highest concordance. Further, these more negatively salient tasks were those that predicted ASD symptomology at 24 months. These observations may provide nuanced differences that can complement standard parent-reported temperament questionnaires.

## Supplementary Information

**Additional file 1.** Brief Coding Scheme for Phases, Affect, and Gaze for the Emotion-Evoking Task.

## Data Availability

The datasets used and/or analyzed during the current study are available from the corresponding author on reasonable request.

## Abbreviations

ADOS: Autism Diagnostic Observation Scale—2nd edition
APA: American Psychiatric Association
ASD: autism spectrum disorder
EE Task: Emotion-Evoking Task
ER: emotion regulation
IBQ-R: Infant Behavior Questionnaire-Revised
IL: infant with an increased likelihood of an ASD diagnosis
Lab-TAB: Laboratory Temperament Assessment Battery
LL: infant with a community-level risk of an ASD diagnosis
