# Supplementary material for: Affect and gaze responses during an Emotion-Evoking Task in infants at an increased likelihood for autism spectrum disorder
Source: Mol Autism. 2021 Oct 6;12:63. doi: 10.1186/s13229-021-00468-0 (PMC8493694; doi:10.1186/s13229-021-00468-0)
Supplement: Supplementary file 1 — Additional file 1. Brief Coding Scheme for Phases, Affect, and Gaze for the Emotion-Evoking Task. [file 13229_2021_468_MOESM1_ESM.docx]

**Additional file 1**

**Table S1:** Brief Coding Scheme for Phases, Affect, and Gaze for the Emotion-Evoking Task

| **Phase**  (continuous, mutually exclusive & exhaustive) | | | | |
| --- | --- | --- | --- | --- |
| **Code** | | **Description** | | |
| Baseline Video 1, 2, or 3 | | From start to end of baseline video: code as number 1, 2 or 3 as they occur | | |
| Bubbles | | Experimenter (E) is blowing bubbles | | |
| Toy Play | | E places toy on table directly in front of infant and allows infant to play | | |
| Toy Removal | | E lifts toy to hold out of reach but in view of infant. | | |
| Blank mask | | E is wearing blank mask | | |
| Cow mask | | E is wearing cow mask | | |
| Wash Face | | E washes infant face with wet wipe | | |
| Brush Hair | | When E is brush infant hair | | |
| Phase Transition/other | | All events outside those described above. | | |
| **Affect**  (instantaneous in 5 s intervals, mutually exclusive and exhaustive) | | | | |
| **Codes** | **Modifiers** | | **Description** | |
| Affect not codable | **-** | | No cues to affect for the entire 5 s interval | |
| Transition | **-** | | 5s interval does not contain coded events | |
| Affect Codable | **2** | | Extreme positive affect | |
|  | **1** | | Mild to moderate positive affect | |
|  | **0** | | Neutral affect | |
|  | **-1** | | Mild to moderate negative affect | |
|  | **-2** | | Extreme negative affect | |
| **Gaze**  (continuous, mutually exclusive & exhaustive) | | | | |
| **Code** | **Description** | | | **Modifier** |
| Parent | Gaze directed to parent’s face or body | | | *At eyes/face* |
|  |  |  |  | *Other* (body) |
|  |  |  |  | *Off screen* |
| Experimenter  (E) | Gaze directed to E’s face or body. This is limited to E conducting task. | | | *At eyes/face* |
|  |  |  |  | *Other* (body) |
|  |  |  |  | Off screen |
| Object | Objects that baby looks at in their immediate environment (on/near table; on their body. This includes the items used for tasks and any other items they may be given/shown). Does not include items distal to the infants that are around the room such as posters, snacks/cups or food they can see or general scanning of the room. | | | *On task*  Task-specific object |
|  |  |  |  | Off task  Objects unrelated to the task; proximal to the infant (on their body or table) but are not task related, e.g., sensors, cups, food, toys unexpectedly given to child by parent |
| Aversion | Infant quickly shifts gaze from presented stimulus/object without clear shift to another object/person, often downwards or slightly to the side and often without head or body movement. Averted gaze will often shift quickly back to presented object and may shift away again in repeated instances of averted gaze. | | | |
| Other | Gaze directed toward an object or area of space in the room other than as defined above. | | | |
| Not codeable | Gaze cannot be coded (eyes shut, crying, facing away from camera) | | | |

**Associations Between IBQ-R at 12 and 18 Months of Age**

The correlations between the IBQ-R subscales at 12 and 18 months were all significant; Activity Level (*r* = .52, *p* < .001); Distress to Limitations (*r* = .65, *p* < .001); Fear (*r* = .46, *p* < .001); Duration of Orienting (*r* = .57, *p* < .001); Smile and Laughter (*r* = .64, *p* < .001); High Pleasure (*r* = .40, *p* = .002); Low Pleasure (*r* = .62, *p* < .001); Soothability (*r* = .57, *p* < .001); Falling Reactivity (*r* = .52, *p* < .001); Cuddliness (*r* = .71, *p* < .001); Perceptual Sensitivity (*r* = .57, *p* < .001); Sadness (*r* = .51, *p* < .001); Approach (*r* = .42, *p* = .001); and Vocal Reactivity (*r* = .71, *p* < .001).

**Presence versus Absence of Positive, Negative, and Neural Evoked Emotion**

We ran two repeated measures ANOVAs to compare the presence or absence of positive, negative, and neutral affect (evoked emotion) during *baseline* and *task* *phases* by group (RL, IL).

***Baseline*.** A repeated measures ANOVA comparing group, sex, age, evoked emotion, and baseline phase found a significant effect for evoked emotion (*F*(2,138)=69.72, *p*<.001), task x evoked emotion (*F*(4,276)= 6.82, *p*<.001), age x evoked emotion (*F*(2,138)=9.89, *p*<.001), phase x age x evoked emotion (*F*(4,276)=8.63, *p*<.001). No other effects or interactions were significant.

***Emotion-Evoking Task*.** A repeated measures ANOVA comparing group, sex, age, evoked emotion, and phase found a significant effect for phase (*F*(6,420)=6.252, *p*<.001), evoked emotion (*F*(2,140)=226.23, *p*<.001), evoked emotion x group (*F*(1,140)=5.05, *p*=.008), and phase x evoked emotion (*F*(12,840)=16.76, *p*<.001). No other effects or interactions were significant.

An examination of the group x evoked emotion interaction showed that overall, the IL group displayed more negative affect (.36) than the LL group (.21; *p* = .016; *d*=.61) throughout the EE Task, with no difference between positive (*p*=.24; *d*=.28) or neutral (*p*=.18; *d*=.33) expressions of affect.

***Incongruent Responding.*** The percentage of children in the LL and IL groups who displayed affect that was incongruent to the probed emotion (e.g., positive response to a negative task or negative response to a positive task, with neutral affect not considered an incongruent response) are shown in Table S2. In general, similar patterns of incongruent responding are seen for both groups, with the exception of hair brushing and face washing, in which the IL group showed few displays of positive affect.  **Table S2.** Percentage of children whose responses were incongruent with the probed emotion

|  | **Bubbles** | **Toy Play** | **Toy Removal** | **Mask 1** | **Mask 2** | **Hair Brush** | **Face Wash** |
| --- | --- | --- | --- | --- | --- | --- | --- |
| **Probed Emotion** | Positive | Positive | Negative | Negative | Negative | Negative | Negative |
| **LL 12 months** | 14.29% | 15.00% | 28.57% | 20.00% | 5.00% | 14.29% | 10.00% |
| **LL 18 months** | 4.76% | 19.05% | 14.29% | 28.57% | 14.29% | 14.29% | 10.53% |
| **IL 12 months** | 21.67% | 21.67% | 20.00% | 23.72% | 18.64% | 1.69% | 3.39% |
| **IL 18 months** | 20.00% | 18.33% | 25.43% | 23.33% | 11.67% | 3.33% | 3.39% |

Note. Cells refer to the percentage of children in the LL (n=21) and IL (n=60) groups who displayed negative affect when presented with a positive task or displayed positive affect when presented with a negative task.
